# Promising prognostic value of Transglutaminase type 2 and its correlation with tumor-infiltrating immune cells in skin cutaneous melanoma

**DOI:** 10.1038/s41420-022-01087-1

**Published:** 2022-06-20

**Authors:** Silvia Muccioli, Roberto Ciaccio, Valentina Brillo, Luigi Leanza

**Affiliations:** Department of Biology, University of Padua, Padua, Italy

**Keywords:** Melanoma, Tumour immunology

## Abstract

Tissue Transglutaminases (TGs) are crosslinking enzymes with pleiotropic functions that have been linked to the development and progression of numerous cancers, with a recent focus on their ability to remodel the tumor microenvironment. Although several pieces of evidence demonstrated their importance in the regulation of the major signaling pathways that control oncogenesis, the correlation between TGs with clinical and pathological features remains controversial and to be further explored. Moreover, an assessment of the TGs alterations together with a functional analysis associated with clinical features and prognostic values are still lacking and would help to understand these intricacies, particularly in human cancers. In the present study, we processed data from numerous public datasets to investigate TGs distribution and prognostic signature in cancer patients. Here, we found that skin cutaneous melanoma (SKCM) shows the highest abundance of TGs mutations among the other human cancers. Interestingly, among all the TGs, TG2 is the only member whose expression is associated with a better overall survival in SKCM, although its expression increases with the worsening of the tumor phenotype. Our analysis revealed a strong positive association between TG2 expression and anti-tumoral immune response, which would explain the relationship between high mRNA levels and better overall survival. Our data suggest that TG2 may be presented as a new promising immune biomarker of prognosis in SKCM, which may contribute to identifying patients who would benefit the most from adjuvant immunotherapy.

## Introduction

Immune system activation in the fight against malignancies has made a significant headway in the last 30 years [1]. Cancer therapies that consist of immune checkpoint inhibitors (ICIs), adoptive T-cell cancer therapy, and cancer vaccination have proven to be useful against tumors immune evasion events, showing remarkable effects on patients’ outcome and overall survival [1].

Skin cutaneous melanoma (SKCM) is one of the most aggressive cancers that arises from transformed melanocytes [2, 3]. Though accounting only for ~1% of skin cancers, SKCM consists of the deadliest subtype, with ~27% of 5-year survival rate for metastatic melanoma according to American Cancer Society 2021 Report (American Cancer Society Cancer Facts & Figures 2021).

Several compounds have been developed and tested in clinical trials in the last 10 years to overcome SKCM high mortality rate, but a treatment for more resistant and refractory tumors still needs to be identified [2]. For this reason, recent melanoma anti-cancer research has mainly been focused on the activation of the host immune system, especially for the possibility of developing specific targeted therapies [3]. Indeed, due to its high mutational load as well as the presence of tumor-associated antigens (as gp100, tyrosinase, and Melan-A) that can elicit immune system activation, SKCM is considered one of the best targets in the field of anti-tumor immunotherapy [3, 4].

However, melanoma response is still limited due to immune evasion mechanisms, such as the production of immune suppressive checkpoint regulators like PD-1/PD-L1 and CTLA-4, of tolerogenic cytokines, and of growth factors released in the tumor microenvironment, which impede maturation, cross-priming, and antigen presentation, altogether leading to progressive loss of immunogenicity [5, 6].

For this reason, shedding light on the molecular mechanisms behind the cross-talk between melanoma and immune cells to favor immune infiltration would improve clinical outcome, as well as identify specific gene signatures for the prognosis of SKCM [6].

In this framework, we decided to evaluate the role of Tissue Transglutaminases (TGs) in SKCM, a family of structurally and functionally related enzymes comprising seven catalytically active isozymes, TG1–7, which all share a common primary structure. TGs carry out various biological functions generally related to the Ca^2+^-dependent post-translational modification of proteins, by introducing covalent bonds between free amine groups (e.g., protein- or peptide-bound lysines) and γ-carboxamide groups of peptide-bound glutamines [7]. TGs family members are widely expressed and can be distinguished based on their physical properties, tissue distribution, localization, and mechanisms of activation, as well as by differences in their substrates and, therefore, in the enzymatic reactions that they catalyze [8].

Among TGs, Transglutaminase type 2 (TG2), the first TG described, is ubiquitously expressed and widespread localized, while other TGs show a tissue related expression [9]. TG2 is mostly cytosolic but can localized also to the plasma and nuclear membranes. In addition, TG2 can be secreted from cells and reside on the cell surface, where it participates to the extracellular matrix remodeling [9]. Traditionally related to several biological processes (e.g., cell signaling, cytoskeleton remodeling, vesicular trafficking, autophagy, and apoptosis) [10], TG2 role in cancer is still debated and controversial. Being involved in drug resistance, metastatic behavior, cancer stem cells potential, inflammation and proliferative signaling, it is often reported to be crucial at all cancer stages [11, 12]. Moreover, novel findings unveiled that TG2 downregulation impairs Wnt-Beta catenin signaling, one of most aberrantly expressed pathways in cancers that also plays a role in immune escape mechanisms [13–15]. Also, TG2 functions in adaptive immune responses; in fact, it was found located on the surface of monocytes and Dendritic Cells (DCs) [16], the principal antigen presenting cells, where it is reported to modulate DC–T-cell interaction [17].

Given this background, we took advantage of transcriptomic data from public databases to carry out a meta-analysis study, which comprehensively analyzed the role of TGs in SKCM. Particularly, by means of mRNA expression signatures, prognostic value analysis, differentially expressed gene ontology analysis, and correlation with tumor-infiltrating immune cells, our research provides information about a correlation between TG2 expression, good SKCM overall survival, and a positive regulation of immune response in SKCM. These results indicate that TG2 expression might serve as prognostic signature in patients with SKCM, as well as a good biomarker for the therapeutic strategy to be adopted, although this notion should be further evaluated in clinical studies.

## Results

### TGs genetic alterations in SKCM and other human cancers

**Fig. 1:**
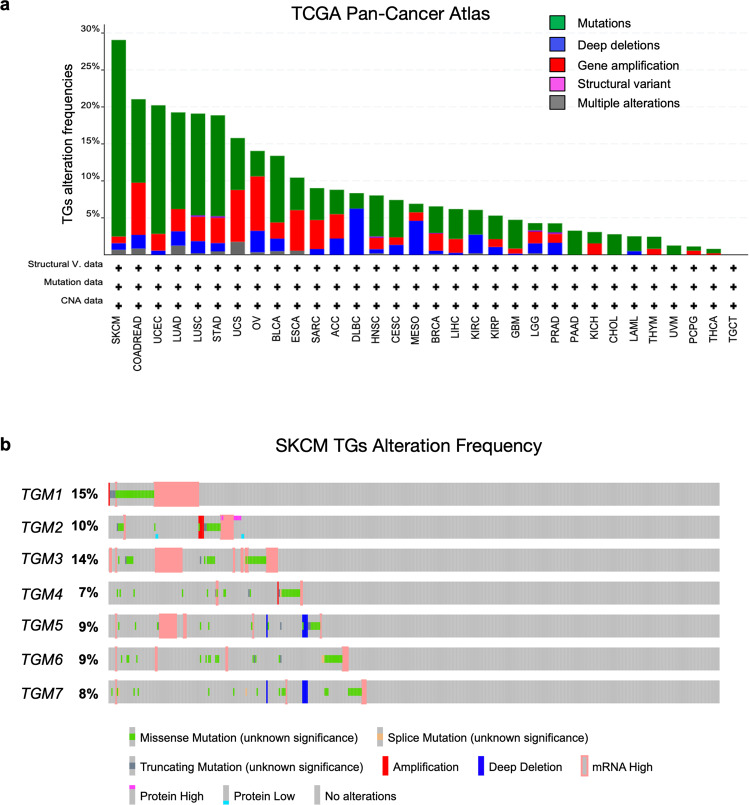
Genomic alteration frequency data of the TGs family in human cancers. **a** Mutation frequencies of TGs in 32 cancer studies were retrieved from cBioPortal (TCGA Pan-Cancer Atlas dataset). **b** Alteration frequencies representation of each TGs member in SKCM dataset were obtained from cBioPortal (TCGA Pan-Cancer Atlas dataset).

To outline an overall view on the status of the TGs genetic alterations in human cancers, we analyzed genetic alteration frequencies of all TGs (*TGM1-TGM7*) and assessed their potential role as prognostic markers using the TCGA Pan-Cancer Atlas dataset, collecting data from 32 human cancers (10967 samples in total). Genomic alterations were classified into five categories as per gene amplification, deep deletions (equivalent to homozygous deletions for non-aneuploidy cases), mutations (truncating mutations, in-frame mutations, or missense mutations), structural variants, and multiple alterations using the cBioPortal database [18]. The cancer type summary representation revealed the distribution of TGs genomic alterations in the PanCancer cohorts (Fig. 1a). Results showed a significantly higher percentage of TGs genomic alterations in SKCM compared to the other analyzed cancer types, with an overall frequency of 29.05%. In particular, 118 SKCM samples showed the presence of truncating mutations, in-frame mutations, or missense mutations in the TGs genes (alteration frequency: 26.58%), while genomic amplification, deep deletions and multiple alterations were present with a frequency of 0.9%, 0.9%, and 0.68% only, respectively (Fig. 1b).

To confirm the high frequency of TGs alterations in SKCM patients, we extended this analysis to other four SKCM datasets: DFCI, Science 2015 [19], DFCI, Nature Medicine 2019 [20], the Melanoma TCGA, Cell 2015 [21], and the Melanoma Yale, Nature Genetics 2012 [22]. Data showed a high frequency of genetic mutations spanning from 13.61% of the Melanoma Yale Dataset to 26.58% of the TCGA one. Interestingly, metastatic melanoma (Van Allen dataset) revealed a consistent level of genomic amplification (4.55%), deep deletions (3.64%) and multiple alterations (1.82%) compared to the other datasets taken into consideration (Fig. S1). Oncoprint representation from cBioPortal was employed to analyze the presence of TGs genomic alterations for each member of the TG gene family. This analysis displayed a similar frequency of genetic alterations for all the seven members of the TG family, spanning from 7% to 15% considering the entire cohort of SKCM patients (Fig. 1b).

### TGs mRNA expression in SKCM and clinical value

**Fig. 2:**
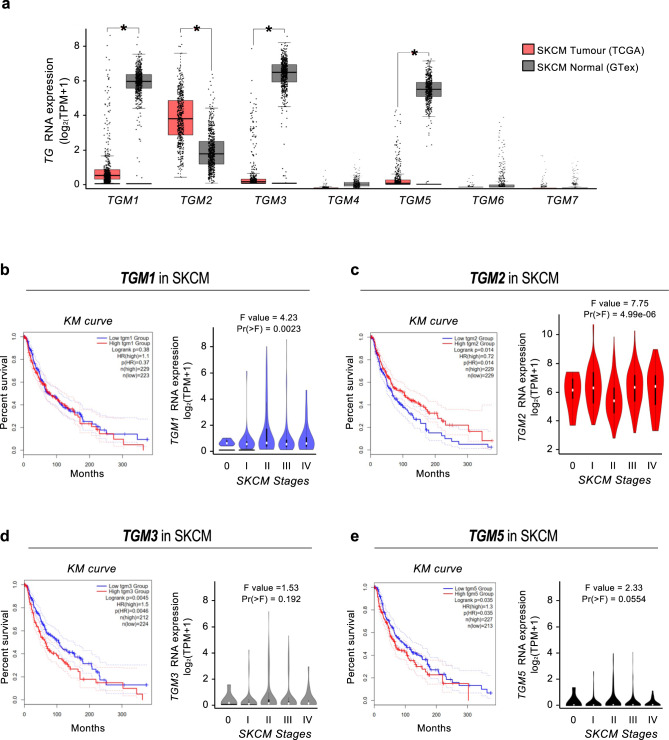
Analysis of the TGs transcriptional levels and clinical value. **a** The mRNA expression of the seven members of the family was assessed comparing tumor and normal tissues from TCGA and GTEx datasets on the GEPIA database. Data were normalized as transcripts per kilobase million (TPM) values. TPM values were converted to log2-normalized transcripts per million [log2(TPM + 1)]. Data were shown as the mean ± standard deviation. Statistical analyses were performed using *t*-test. Error bars represented SD. *, **, *** indicated *p*-value < 0.05, 0.01, 0.001, respectively. **b**–**e** Overall survival based on *TGM1*, *TGM2*, *TGM3*, and *TGM5* expression level in SKCM was obtained through Kaplan–Meier analysis by sorting samples for high and low TG expression groups according to the median on GEPIA, as well as the expression of these TGs in the different SKCM stages. Percent survival was plotted, and *p*-values were shown as per figure specifications, respectively. *TGM1, TGM2, TGM3,* and *TGM5* mRNA expression was stratified as per SKCM stages. Values were normalized as transcripts per kilobase million (TPM) values. TPM values were converted to log2-normalized transcripts per million [log2(TPM + 1)]. Statistical analyses were performed using Fisher’s exact test. *p*-values were indicated, respectively.

The investigation of the clinical significance of TGs in patients affected by SKCM was then evaluated using the publicly available GEPIA database (Gene Expression Profiling Interactive Analysis) [23]. We compared mRNA expression levels in both tumor and normal samples. Our analysis revealed increased expression levels of *TGM1*, *TGM3*, and *TGM5* genes in normal samples compared to tumors. Interestingly, *TGM2* mRNA levels showed an opposite trend, presenting a significantly higher expression in SKCM neoplastic samples compared to controls, exhibiting the highest mRNA expression levels upon all the TGs in SKCM cancer samples. In addition, *TGM4*, *TGM6*, and *TGM7* genes were the lowest expressed and did not exhibit statistically significant differences in the level of expression between tumor and healthy tissues (Fig. 2a). To better investigate the association between *TGM1*, *TGM2*, *TGM3*, and *TGM5* expressions and the overall survival in the same cohort of SKCM patients, we made use of the Kaplan–Meier analysis by sorting samples for high and low TGs expression groups according to the median TGs mRNA levels. In addition, we also evaluated the expression levels of the same genes in all SKCM stages to take into consideration cancer aggressiveness. These analyses confirmed the prognostic value of *TGM2*, *TGM3*, and *TGM5* but not *TGM1* in the pathology. Indeed, no statistically significant differences were highlighted between high and low *TGM1* expressing samples in terms of survival (Log-rank *p* = 0.38) and expression in the five different SKCM stages (*F*-value = 4.23; Pr(>*F*) = 0.0023) (Fig. 2b). Interestingly, high *TGM2* expression appears significantly associated with an increase in terms of survival rate (Log-rank *p* = 0.014), but it was also found to be significantly and positively associated with the SKCM tumor stages (*F*-value = 7.75; Pr(>*F*) = 4.99e-06) (Fig. 2c). Differently, high *TGM3* and *TGM5* mRNA expression correlates with worst overall survival, although gene expression levels appear remarkably lower compared to both *TGM1* and *TGM2* genes (Fig. 2d, e). Given these premises, we sought to investigate the role of *TGM2* among all the TGs in SKCM neoplasia in more detail, to better characterize its positive prognostic role in this pathology.

### Clinical features and prognostic value of *TGM2* in SKCM

**Fig. 3:**
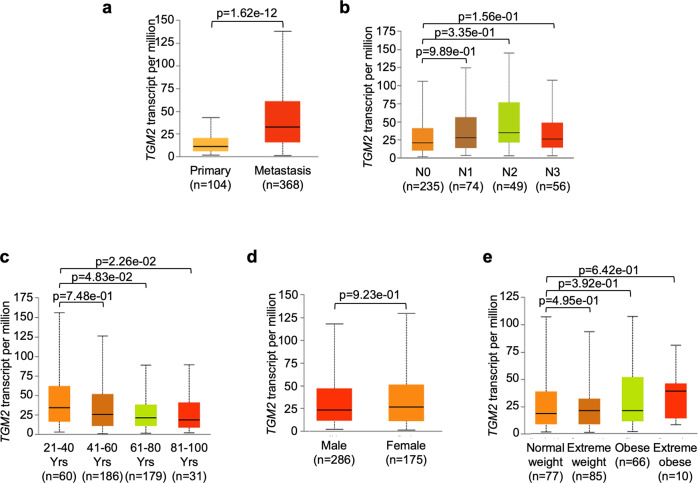
Clinical characterization and prognostic value of TGM2. Boxplots represent the relationship between *TGM2* mRNA expression with the absence or presence of tumor metastasis (**a**), nodal metastasis formation from stage N0 to N3 (**b**), age (**c**), sex (**d**), and body mass index (BMI) (**e**) in patients affected by SKCM using the TCGA database on UALCAN. Data were normalized as transcripts per kilobase million (TPM) values. Data were shown as the mean ± standard deviation. Statistical analyses were performed using t-test. Error bars represented SD. *p*-values were indicated, singularly.

To explore *TGM2-*related clinical characters, we determined the relationship between the *TGM2* expression level in SKCM and pathological clinical features. Therefore, we investigated whether *TGM2* expression correlates with the absence or presence of tumor metastasis, age, sex, and body mass index (BMI) in patients affected by this pathology using the UALCAN database [24]. Interestingly, the boxplots indicate that transcription levels of *TGM2* in SKCM patients were approximately three-fold higher in metastatic samples compared to the primary ones (*p* = 1.62e-12) (Fig. 3a). In addition, we also found a significant increment in *TGM2* mRNA levels in samples with increasing abnormality of lymph nodes (size, characteristics, location) and nodal metastasis compared to control (Fig. 3b). As for all kinds of cancer, the incidence of melanoma advances with age, since age is also a crucial predictive factor in SKCM, as increasing age showed to worsen survival in stages I, II and III [25]. Moreover, Sentinel lymph node (SLN) status, which is a strong predictor of melanoma survival, is also affected by age, as SLN positivity decreases with age [26]. We found that *TGM2* mRNA expression levels were significantly lowered in older patients compared to the younger ones (Fig. 3c). We also found that *TGM2* mRNA levels of expression were significantly higher in females compared to male samples (*p* = 9.23e-01) (Fig. 3d), and that *TGM2* levels correlate with patients’ body weight, being significantly higher in extreme weight (*p* = 4.95e-01), obese (*p* = 3.92e-01), and extreme obese patients (*p* = 6.42e-01) compared with normal-weight ones (Fig. 3e).

### Identification and analysis of *TGM2* related differentially expressed genes

**Fig. 4:**
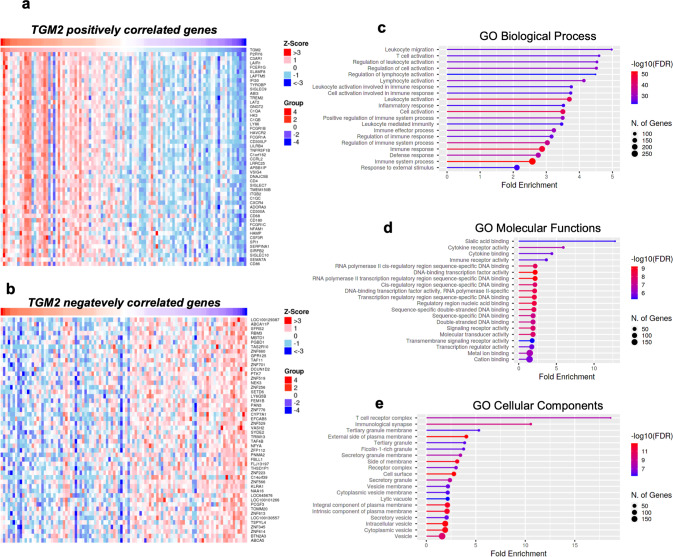
TGM2 correlated differentially expressed genes and related pathways. **a**, **b** The top 50 positively and top 50 negatively *TGM2* co-expressed genes were mapped using the TCGA SKCM dataset in the LinkedOmics database, according to their ranking based on their *Z*-score through Pearson Correlation analysis. **c**–**e** GO analysis obtained through Gene Ontology Enrichment Analysis performed on ShinyGO, using the top 1000 *TGM2* positively correlated genes based on their *p*-value in SKCM (BP, biological process; CP, cellular component; GO, Gene Ontology; MF, molecular function). Data were plotted as per fold enrichment, for the -log10 of the false-discovery rate (FDR) and the *p*-values, respectively.

To further assess *TGM2* role in SKCM, we sought to identify *TGM2*-related differentially expressed genes (DEGs) and performed Gene Ontology analysis (GO) to clarify the pathways they are involved in. Particularly, the top fifty positively (Fig. 4a) and the top fifty negatively (Fig. 4b) *TGM2* correlated genes were ranked based on their Z-score and mapped using the TCGA SKCM dataset in LinkedOmics database [27]. Interestingly, many genes known to be associated with the activation of immune-related cancerous processes, response to immunotherapy, and tumor immune infiltration were found to be associated with *TGM2* expression like *C3AR1* (*r* = 0.5712, *p* = 2.956e-10) [28], *LAIR1* (*r* = 0.5612, *p* = 7.015e-10) [29], *FCER1G* (*r* = 0.5588, *p* = 0,8553e-10) [30], *SLAMF8* (*r* = 0.5562, *p* = 1.066e-10) [31], *IFI30* (*r* = 0.5472, *p* = 2.228e-10) [32], *TYROBP* (*r* = 0.5462, *p* = 2.416e-09) [33], and *SIGLEC-9* (*r* = 0.5442, *p* = 2.829e-09) [34], which were found in the top ten *TGM2* positively correlated ones through two-sided Pearson’s (*R*) coefficient analysis (Fig. S2).

As a further confirmation of the correlation between *TGM2* expression and tumor immune infiltration, we performed Gene Ontology (GO) enrichment analysis, using the top 1000 *TGM2* positively correlated targets, which revealed a strong association with immune system activation and immune response activity (Fig. 4c–e). Particularly, most of the categories enriched in the GO Biological Processes are remarkably involved in immune response activation, like “Leukocyte migration”, “T-cell activation”, “positive regulation of immune system process”, and “defense response” (Fig. 4c). On the same line, both GO Cellular Components and GO Molecular Functions analyses confirmed the implication in immune system regulation, being “T-cell receptor complex” and “Sialic acid-binding” the respective top-ranking categories (Fig. 4d, e).

### Correlation between *TGM2* expression and tumor immune microenvironment activation in SKCM

**Fig. 5:**
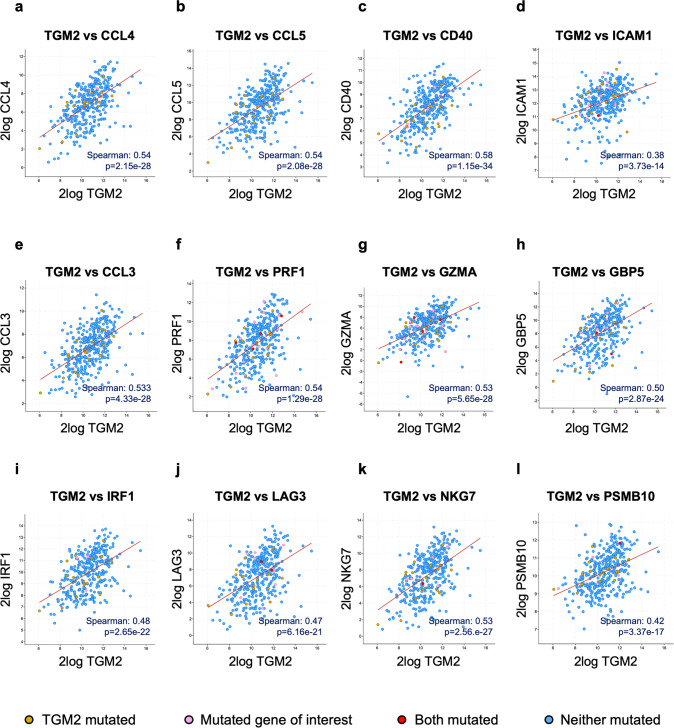
TGM2 correlation with immune activation markers. **a**−**l** Two Pearson correlation analysis between *TGM2* level of expression and immune activation markers were obtained from cBioPortal using the SKCM TCGA PanCancer Atlas dataset. Data were plotted as scatter plot based on the Spearman Correlation Coefficient (363 samples) (*p < 0.05; **p < 0.01; ***p < 0.001) and stratified as per *TGM2* mutations (gold), correlated gene of interest’s mutations (pink), absence of mutations (blue), presence of both mutations in the same sample (red).

Tumor-infiltrating immune cells play a critical role in the fight against cancer. Indeed, immune system response to melanoma requires a set of actions and interactors that must anticipate and counteract immune escape mechanisms adopted by the tumor. In this concerted process, even the tumor itself, by accumulating immunogenic mutations or expressing certain genes, contributes to triggering the activation of immune cells [35]. Therefore, a correlation analysis of *TGM2* expression with some of the genes involved in the prognosis of SKCM, immune system activation, and immunotherapy response was pursued with cBioPortal. Results showed that *TGM2* expression exhibited a positive and significant relationship with numerous already known immune activation markers, which are reported in detail in Table 1. One crucial player in this mechanism are dendritic cells (DCs), which are known to have effector activities in redirecting cytotoxic CD8^+^ T cells against the tumor by antigen-cross presentation [36]. Several are the markers of the recruitment of such immune cell type, mainly chemokines like CCL4 (Fig. 5a), CCL5 (Fig. 5b), receptors and ligands as CD40 (Fig. 5c), and several growth factors, which are all reported to be strongly co-expressed with *TGM2* in SKCM. Tumor-associated neutrophils (TANs) have also a role in cancer immune response. Indeed, specific stimulating chemokines and factors released in the tumor microenvironment lead to the polarization of TANs to anti-tumor (N1) or pro-tumor (N2) phenotype [37]. Immune profile of N1 TANs is elicited by high levels of ICAM-1 (Fig. 5d) and CCL3 (Fig. 5e) [37], which also correlated with *TGM2* expression in SKCM.

**Table 1:** *TGM2* expression and tumor immune microenvironment activation in SKCM.

Immune cell type	Gene markers	Spearman’s correlation	*p*-value	Ref.
General T-cell activation	CD3D	**0.521**	**1.34e-26**	[55]
	CD3E	**0.539**	**8.58e-29**	[56]
CD4 + T cells (Th1 and Th2)	TBX21	**0.509**	**2.44e-25**	[57]
	HLA-DMB	**0.595**	**3.77e-36**	[46]
	HLA-DRA	**0.567**	**2.58e-32**	[47]
	TGF- β	**0.499**	**3.32e-24**	[48]
	CRTAM	**0.524**	**5.46e-27**	
	IL12A	**0.237**	**5.083e-6**	
	IL12B	**0.381**	**5.11e-14**	
Dendritic cells (DCs)	VEGFC	**0.517**	**3.36e-25**	[58]
	CCL4	**0.536**	**2.15e-28**	[36]
	CCL5	**0.536**	**2.08e-28**	
	CD40L	**0.455**	**6.18e-20**	[59]
	CD40	**0.585**	**1.15e-34**	
Neutrophils (N1 population)	CXCL5	**0.329**	**1.26e-10**	[60]
	CSF2	**0.391**	**9.82e-15**	[61]
	CSF3	**0.261**	**4.67e-7**	
	CCL3	**0.533**	**4.33e-28**	[37]
	TNFα	**0.432**	**6.70e-18**	
	ICAM1	**0.383**	**3.73e-14**	
	TNFRSF4	**0.455**	**5.76e-20**	[62]
	CXCL10	**0.341**	**2.47e-11**	[63]
	CCL5	**0.536**	**2.08e-28**	[38]
	PRF1	**0.538**	**1.29e-28**	
	GBP5	**0.499**	**2.87e-24**	
	GZMA	**0.533**	**5.65e-26**	
	IRF1	**0.480**	**2.65e-22**	
	LAG3	**0.466**	**6.16e-21**	
	NKG7	**0.527**	**2.56e-27**	
	PSMB10	**0.423**	**3.37e-17**	
Multiple immune cells activation (in melanoma)	IL21	**0.394**	**6.59e-15**	[64, 65]
	IFNG	**0.453**	**8.69e-20**	[61]
	CXCL13	**0.429**	**1.03e-17**	[66]

Correlation analysis was carried out by Two Pearson correlation analysis between *TGM2* level of expression and the presented immune activation markers using cBioPortal SKCM TCGA PanCancer Atlas dataset (363 samples).

Moreover, in 2021 Yan et al. identified eight biomarkers expressed by SKCM cells that specifically recruit and activate CD8 + T cells to promote cytotoxic activity [38]. All these good prognostic biomarkers—CCL5 (Fig. 5b), PRF1 (Fig. 5f), GZMA (Fig. 5g), GBP5 (Fig. 5h), IRF1 (Fig. 5i), LAG3 (Fig. 5j), NKG7 (Fig. 5k), and PSMB10 (Fig. 5l)—are all positively correlating with *TGM2* expression levels in SKCM.

Several other markers associated with the promotion of immune infiltration in tumor microenvironment that are linked to *TGM2* expression are also reported in Table 1. Collectively, these results indicate that *TGM2* expression in SKCM may mediate immune system activation on multiple levels.

### High *TGM2* expression is associated with increased immune infiltration

**Fig. 6:**
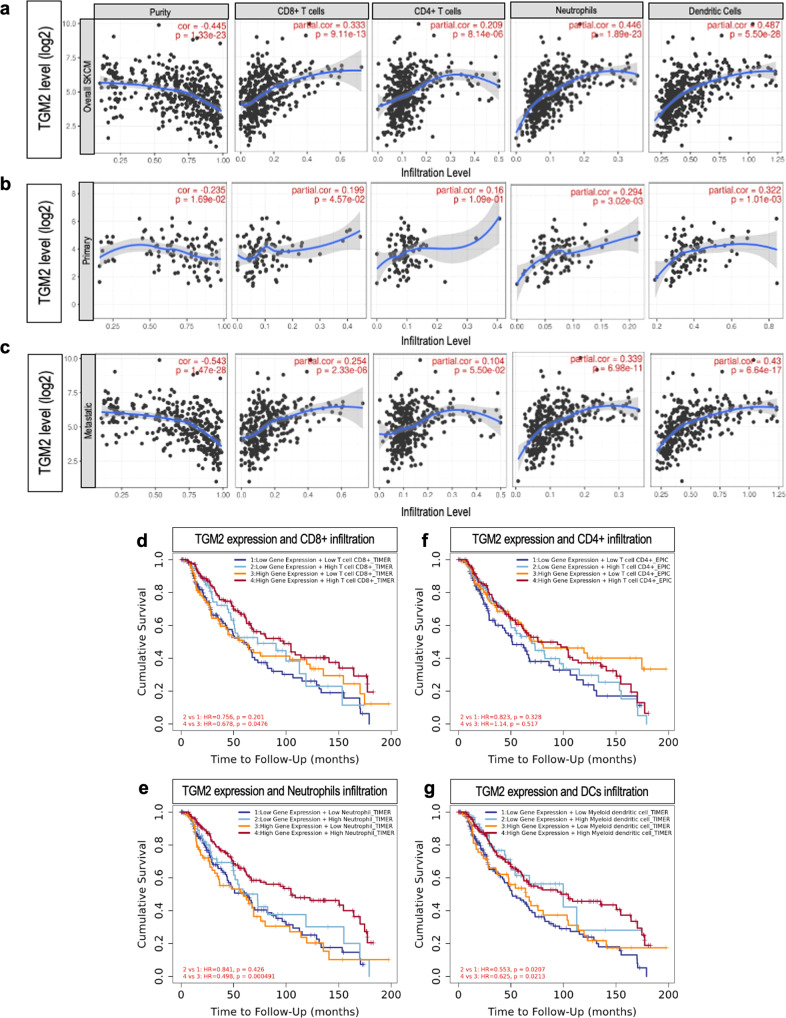
TGM2 correlation with infiltrating immune cells and prognostic value in SKCM. **a**–**c** Correlation between *TGM2* level of expression and immune infiltration cell types was obtained in overall, primary and metastatic SKCM. **d**−**g** Overall survival analysis was retrieved through Kaplan–Meier by sorting samples for high and low *TGM2* level of expression and immune cell types infiltration in SKCM, according to their median. These analyses were performed using TIMER (*p < 0.05; **p < 0.01; ***p < 0.001). Fifteen panels, showing the purity-corrected partial Spearman correlation and statistical significance between *TGM2* expression and each SKCM subset, were displayed.

We next investigated whether *TGM2* expression is related to immune infiltration levels in SKCM. To achieve this purpose, we took advantage of the Tumor Immune Estimation Resource (TIMER) database, a comprehensive resource platform that can access the systematic analysis of immune infiltrates in various types of cancers [39]. We evaluated the abundance of six immune cell populations in the tumor microenvironment of the SKCM patients collected inside the TCGA cohorts: B cells, CD4 + T cells, CD8 + T cells, Neutrophils, Macrophages, and Dendritic Cells. Specifically, we analyzed the gene expression profiles measured through RNA-seq in SKCM (Fig. 6a), Primary SKCM (Fig. 6b) and Metastatic SKCM (Fig. 6c) to investigate the influence of *TGM2* expression in SKCM cells impacting on the immune response process. Tumor purity is a key element affecting the genomic analysis of immune infiltrates and, in this case, all the screened conditions showed negative correlations with this parameter. In whole SKCM, positive correlations were found between *TGM2* levels and all the screened immune cell types: CD8 + T cells (*r* = 0.333, *p* = 9.11e-13), CD4 + T cells (*r* = 0.209, *p* = 0.814e-06), neutrophils (*r* = 0.446, *p* = 1.89e-23), dendritic dells (*r* = 0,487, *p* = 5.50e-28) (Fig. 6a), B cells (*r* = 0.225, *p* = 1.44e-06), and macrophages (*r* = 0.439, *p* = 8.73e-23), (Fig. S3a). Interestingly, the associations of *TGM2* expression with immune cell infiltration varied evidently between primary SKCM (Fig. 6b and Fig. S3b) and metastatic SKCM (Fig. 6c and Fig S3c). In primary SKCM, *TGM2* expression leads to a lower association with immune cell infiltration in all the six cell types taken into consideration. By contrast, metastatic SKCM showed an increased association compared to the primary one, suggesting that *TGM2* might be related to tumor immune infiltration in SKCM and that this association is more pronounced in the metastasis. In addition, we also provided evidence that the cumulative survival of SKCM patients significantly improves when a higher expression of the *TGM2* gene is associated with a stronger infiltration of CD8 + T cells (Fig. 6d), Neutrophils (Fig. 6e), and Dendritic Cells (Fig. 6g), compared to the increase in tumor-infiltrating immune cells alone. However, no significative changes in overall survival are appreciable when a high TGM2 expression is associated with a greater infiltration of CD4 + T cells (Fig. 6f), B cells (Fig. S3d), and macrophages (Fig. S3e).

## Discussion

SKCM is a highly immunogenic cancer whose interplay with immune resident and infiltrating cells in the microenvironment significantly impacts on proliferation, disease progression, and metastasis formation [5]. Indeed, highly proliferative melanoma cells release a wide variety of tolerogenic factors and interleukins to create an immunosuppressive milieu and adopt immune evasion strategies [5]. For this reason, immunotherapy has proven to be effective in SKCM treatment even in advanced stages, by employing, for example, the Immune Checkpoint Inhibitors (ICIs), which totally revolutionized melanoma treatment in the last decades [40].

However, long-term responses to immunotherapy concern only a minority of melanoma patients (30-50%) [40, 41]. Thus, on the one hand it is necessary to find predictive biomarkers of immune response to immunotherapy, while on the other hand, alternative treatment approaches are required to elicit immune system response, like ameliorating tumor antigen presentation and boosting cytotoxic-T-cells pool in cancer stroma [40].

Our meta-analysis study shows that among all the TGs, which are mutated up to the 50% of SKCM cases, TG2 could have a protective though controversial role. Indeed, even though its expression increases in metastatic and advanced stage cancers, high levels of TG2 are associated with a better overall survival rate in SKCM, as demonstrated by the Kaplan–Meier analysis. TG2 is the most ubiquitously expressed and well characterized member of the TGs, whose role is still highly debated, mainly due to its multifunctionality [9]. Described as both an anti- [42] and pro-apoptotic factor [43], very few and controversial results are reported in literature about TG2 role in metastatic melanoma, principally regarding its ability to remodel tumor microenvironment niche [42, 43].

To shed light on this issue, using publicly available mRNA expression data we identified TG2-related differentially expressed genes (DEGs) in SKCM and performed Gene Ontology Analysis to find the main pathways whose expression is associated with that of TG2. Interestingly, our results consistently demonstrate that a higher TG2 expression correlates with the activation of immune system response. We also show that this regulation is articulated through a concerted action between dendritic cells, CD4 + helper, Neutrophils, and cytotoxic CD8 + T-cells. In fact, on the one hand, factors that positively correlate with TG2 such as CCL4 and CCL5 chemokines, VEGF-C, and CD40 ligand all contribute to the recruitment of dendritic cells (DCs), which are known to promote cytotoxic CD8 + T-cells infiltration in tumor microenvironment by antigen-cross presentation, enhancing patients’ survival and response to ICIs [44]. On the other hand, the expression of several HLA major histocompatibility complex class II molecules and chemokines that are known to foster immune infiltration by recalling CD4 + T helpers, eliciting response to ICIs, and ameliorating patients’ prognosis, also correlate with TG2 expression [45]. Among them, HLA-DMB [46], HLA-DRA [47], IL12A, and IL12B [48], whose determinant role in cancer outcome as immune adjuvants has already been published, are found in the top 500 TG2 positively correlating genes. Finally, also TAN N1 stimulating genes (e.g., TNFα, ICAM-1, and CCL3) are strongly co-expressed with *TGM2*.

Links between TG2 and the activation of immune system response are already mentioned in literature: for example, TG2 was reported to be involved in dendritic cells and monocyte maturation [49], as well as participating in dendritic cell-T-cell docking [17]. Also, very recently [50] revealed that TG2 regulates innate immunity via the STING/TBK1/IRF3 axis.

Given these assumptions, we wanted to confirm whether TG2 expression truly correlates with the infiltration of various immune infiltrating cell types in SKCM on TIMER database. Intriguingly, our analysis revealed that the more the tumor is metastatic, the stronger the correlation between immune system infiltration and TG2 expression is, with a direct outcome on survival rate curves. Thus, a higher expression of TG2 may determine a release of molecules attractive towards the immune system, which could in turn explain the better survival rate of SKCM patients, even in the most aggressive forms of this neoplasia.

In conclusion, we believe that our work could pave the way for better assessing TG2 role in SKCM. Indeed, we provided evidence that not only TG2 can be considered a good prognostic marker in melanoma, but also that its expression could affect tumor interplay with the microenvironment via regulating infiltrating immune cells, so that TG2 may be taken into account as a possible predictive immunotherapeutic biomarker in SKCM. As already demonstrated in *Schisotosoma*-induced liver fibrosis [51], further experimental work is required to pinpoint the molecular link between TG2 expression and the release of soluble factors able to recruit specific immune cells into the tumor microenvironment with beneficial effects.

## Materials and methods

### Data collections: TCGA (The cancer genome atlas) and GTEx (Genotype-tissue expression)

TCGA is a freely web-based accessible database, which collects NGS data from more than 11,000 tumors across 33 cancer types until 2018, providing gene expression, methylation, copy number variation datasets, and clinical data [52]. Genotype-tissue expression (GTEx) GTEx provides publicly available gene expression data from 53 normal tissue sites across nearly 1000 people by RNA sequencing [53].

### cBioPortal

The cBioPortal for Cancer Genomics (http://www.cbioportal.org) is a repository of cancer genomics datasets [18, 54]. We investigated genetic alteration frequencies of all TGs (*TGM1-TGM7*) and assessed their potential role as prognostic markers using the TCGA Pan-Cancer Atlas dataset, collecting data from 32 human cancers (10967 samples in total) and four SKCM datasets: DFCI, Science 2015 [19], DFCI, Nature Medicine 2019 [20], the Melanoma TCGA, Cell 2015 [21], and the Melanoma Yale, Nature Genetics 2012 [22].

### Gene expression profiling interactive analysis (GEPIA)

GEPIA (gene expression profiling interactive analysis) (http://gepia.cancer-pku.cn/) is an interactive web application for gene expression analysis based on 9736 tumors and 8587 normal samples [23]. GEPIA was used to analyze the expression of TGs in SKCM and their effects on survival rate by means of the Kaplan–Meier analysis tool. We divided samples between high and low TGs expression groups according to the median TGs mRNA levels to analyze overall survival (Log2FC cutoff: 1; *p*-value cutoff: 0.01, group cutoff selected median, cutoff-high (%): 50; cutoff-low (%): 50).

### UALCAN database analysis

To determine the clinical value of TG2 expression level, the UALCAN database (http://ualcan.path.uab.edu) was employed. UALCAN is an interactive web resource for analyzing cancer OMICS data and correlate them with clinicopathological features [24]. UALCAN was used to evaluate the correlation between TG2 level of expression and several clinical signatures like the absence or presence of tumor metastasis, nodal metastasis status, age, sex, and weight in patients affected by SKCM. Nodal metastasis status was classified in: N0 (no regional lymph node metastasis), N1 (metastasis in 1 to 3 axillary lymph nodes), N2 (metastasis in 4 to 9 axillary lymph nodes), and N3 (metastasis in 10 or more axillary lymph nodes). Patients’ weight was stratified based on BMI (body mass index): Normal weight (18.5 ≤ BMI ≤ 25), Extreme weight (25 ≤ BMI ≤ 30), Obese (30 ≤ BMI ≤ 40), and Extreme obese (BMI ≥ 40).

### Tumor immune estimation resource (TIMER)

TIMER (tumor immune estimation resource) is an in silico user-friendly website to analyze interactions between tumor and infiltrating immune components [39]. This website has incorporated samples from 32 different kinds of cancer types from the TCGA dataset, gathering gene expression profiles measured with RNA-seq or microarray to evaluate the abundance of different immune cell types in the TME. TG2 expression and its association with infiltrated immune cells (B cells, CD4 + T cells, CD8 + T cells, Neutrophils, Macrophages, and Dendritic Cells) was assessed in overall, primary, and metastatic SKCM.

### LinkedOmics

The LinkedOmics database (http://www.linkedomics.org/login.php) is a platform for analyzing 32 TCGA cancer-associated multi-dimensional datasets [27]. The LinkFinder module of LinkedOmics was used to study differentially expressed genes in correlation with TG2 in the TCGA SKCM Firehorse cohort (*n* = 371) and to perform pathway and network analyses. Results were analyzed statistically using Pearson’s correlation coefficient. GO enrichment analysis was used to perform GO analyses (CC, BP and MF) by employing the ShinyGO online software (DOI: 10.1093/bioinformatics/btz931).

### Statistical analysis

Analyses for statistical interpretation were performed using the CbioPortal, GEPIA, UALCAN, LinkedOmics, and TIMER databases. Differences were examined for significance as per figure legend specifications. Data were normalized as transcripts per kilobase million (TPM) values. In Fig. 2, we converted the TPM values in log2-normalized transcripts per million [log2(TPM + 1)]. Data were shown as the mean ± standard deviation. Statistical analyses were performed using *t*-test. Error bars represented SD. *, **, *** indicated *p*-value < 0.05, 0.01, 0.001, respectively.

Kaplan–Meier analyses were plotted based on Fisher’s exact test (*F*-test). *TGM1, TGM2, TGM3,* and *TGM5* mRNA expression levels were stratified as per SKCM stages. Values were normalized as TPM. Statistics of the survival analyses were performed using *F*-test. The top 50 positively and top 50 negatively *TGM2* co-expressed genes were mapped using the TCGA SKCM dataset in the LinkedOmics database, according to their ranking, based on their *Z*-score through Pearson Correlation analysis. The top 1000 *TGM2* positively correlated genes were used for GO analysis obtained through Gene Ontology Enrichment Analysis performed on ShinyGO, based on their *p*-value in SKCM. Data were plotted as per fold enrichment, for the –log10 of the false-discovery rate (${\mathrm{FDR}} = 1 - \frac{{Nk}}{T}$), and the *p*-values, respectively. Two Pearson correlation analyses between *TGM2* level of expression and immune activation markers were obtained from cBioPortal using the SKCM TCGA PanCancer Atlas dataset. Data were plotted as scatter plot based on the Spearman Correlation Coefficient (363 samples) (*p < 0.05; **p < 0.01; ***p < 0.001). On the other hand, correlation between *TGM2* level of expression and immune infiltration cell types was obtained in overall, primary, and metastatic SKCM. Fifteen panels, showing the purity-corrected partial Spearman correlation and statistical significance between *TGM2* expression and each SKCM subset, were displayed.

## Supplementary information

Supplementary materialś

## Data Availability

All data are available in the main text or the supplementary materials.
