# Supplementary material for: Promising prognostic value of Transglutaminase type 2 and its correlation with tumor-infiltrating immune cells in skin cutaneous melanoma
Source: Cell Death Discov. 2022 Jun 20;8:294. doi: 10.1038/s41420-022-01087-1 (PMC9209462; doi:10.1038/s41420-022-01087-1)
Supplement: Supplementary file 1 — Supplementary materialś [file 41420_2022_1087_MOESM1_ESM.pdf]

SUPPLEMENTARY INFORMATION

Promising prognostic value of Transglutaminase type 2 and its correlation with tumor-infiltrating immune cells in Skin Cutaneous Melanoma

Silvia Muccioli[1*], Roberto Ciaccio[1*], Valentina Brillo[1], Luigi Leanza[1#]

Figure S1. Genomic alteration frequency data of the TGs family in various SKCM datasets. a) Mutation frequency of TGs in 5 skin cutaneous melanoma cancer studies were accessed from cBioPortal.

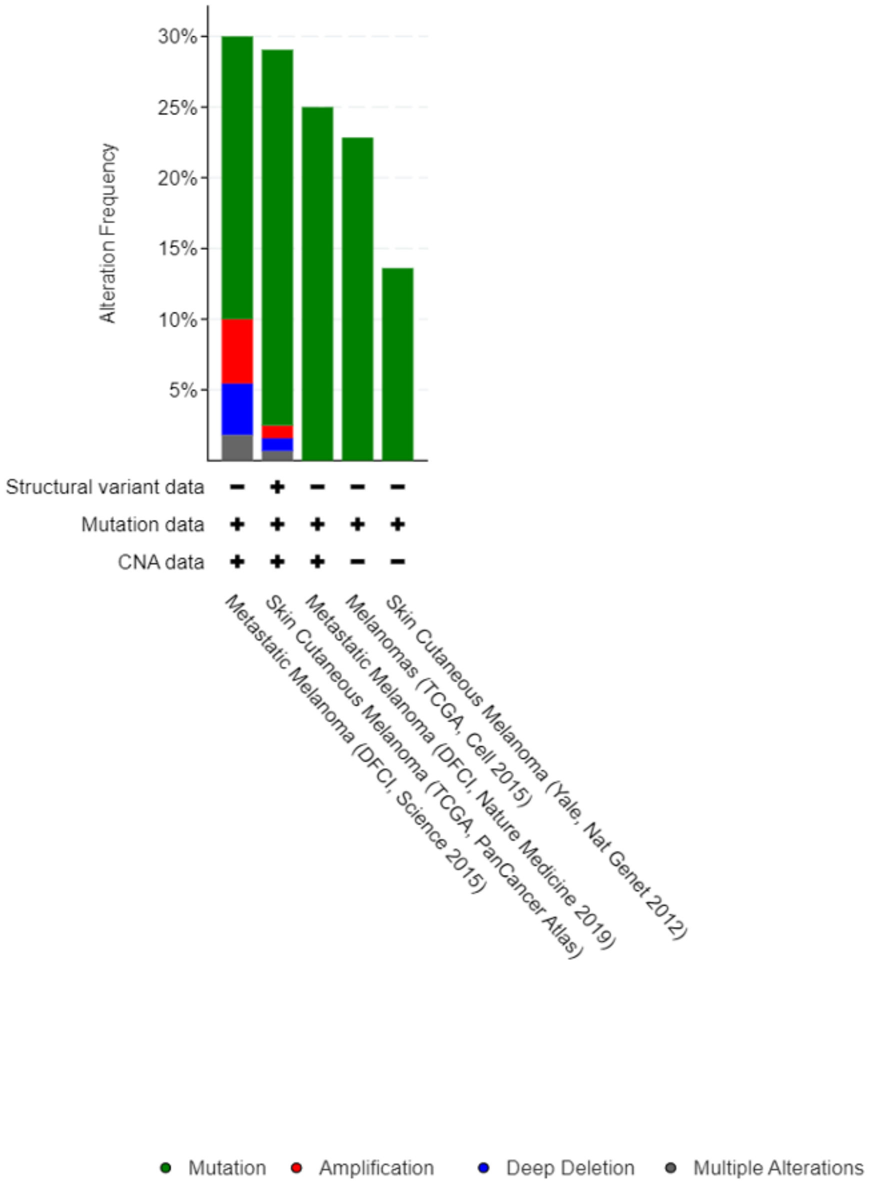

Alteration Frequency
30%
25%
20%
15%
10%
5%
Structural variant data
Mutation data
CNA data
Metastatic Melanoma (DFCI, Science 2015)
Skin Cutaneous Melanoma (TCGA, PanCancer Atlas)
Metastatic Melanoma (DFCI, Nature Medicine 2019)
Melanomas (TCGA, Cell 2015)
Skin Cutaneous Melanoma (Yale, Nat Genet 2012)
Mutation
Amplification
Deep Deletion
Multiple Alterations

Figure S1

Figure S2. Correlation between TGM2 level of expression with genes involved in cancer immune response. Two Pearson correlation analysis were obtained from LinkedOmics (*p<0.05; **p<0.01; ***p<0.001).

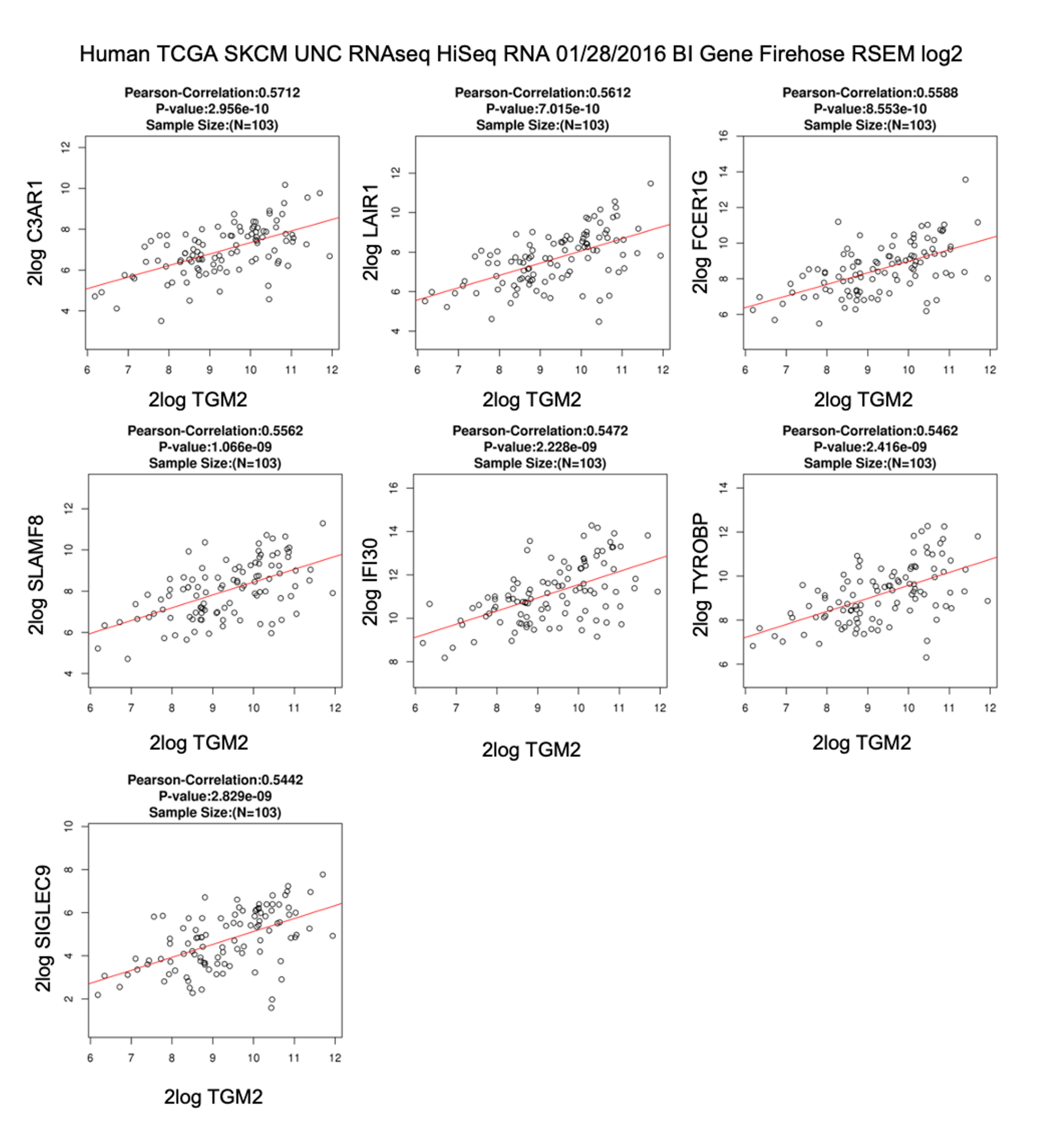
Human TCGA SKCM UNC RNAseq HiSeq RNA 01/28/2016 BI Gene Firehose RSEM log2
Pearson-Correlation:0.5712
P-value:2.956e-10
Sample Size:(N=103)
2log C3AR1
2log TGM2
Pearson-Correlation:0.5612
P-value:7.015e-10
Sample Size:(N=103)
2log LAIR1
2log TGM2
Pearson-Correlation:0.5588
P-value:8.553e-10
Sample Size:(N=103)
2log FCER1G
2log TGM2
Pearson-Correlation:0.5562
P-value:1.066e-09
Sample Size:(N=103)
2log SLAMF8
2log TGM2
Pearson-Correlation:0.5472
P-value:2.228e-09
Sample Size:(N=103)
2log IFI30
2log TGM2
Pearson-Correlation:0.5462
P-value:2.416e-09
Sample Size:(N=103)
2log TYROBP
2log TGM2
Pearson-Correlation:0.5442
P-value:2.829e-09
Sample Size:(N=103)
2log SIGLEC9
2log TGM2

Figure S2.

Figure S3. *TGM2* correlation with infiltrating B cells and macrophages and their prognostic value in SKCM. (a-c) Correlation between *TGM2* level of expression and immune infiltration of B cells and macrophages was obtained in overall, primary and metastatic SKCM. (d-e) Overall survival analysis was retrieved through Kaplan Meyer by sorting samples for high and low *TGM2* level of expression and immune cell types infiltration in SKCM, according to their median. These analyses were performed using TIMER (*p<0.05; **p<0.01; ***p<0.001).

Fig. S3

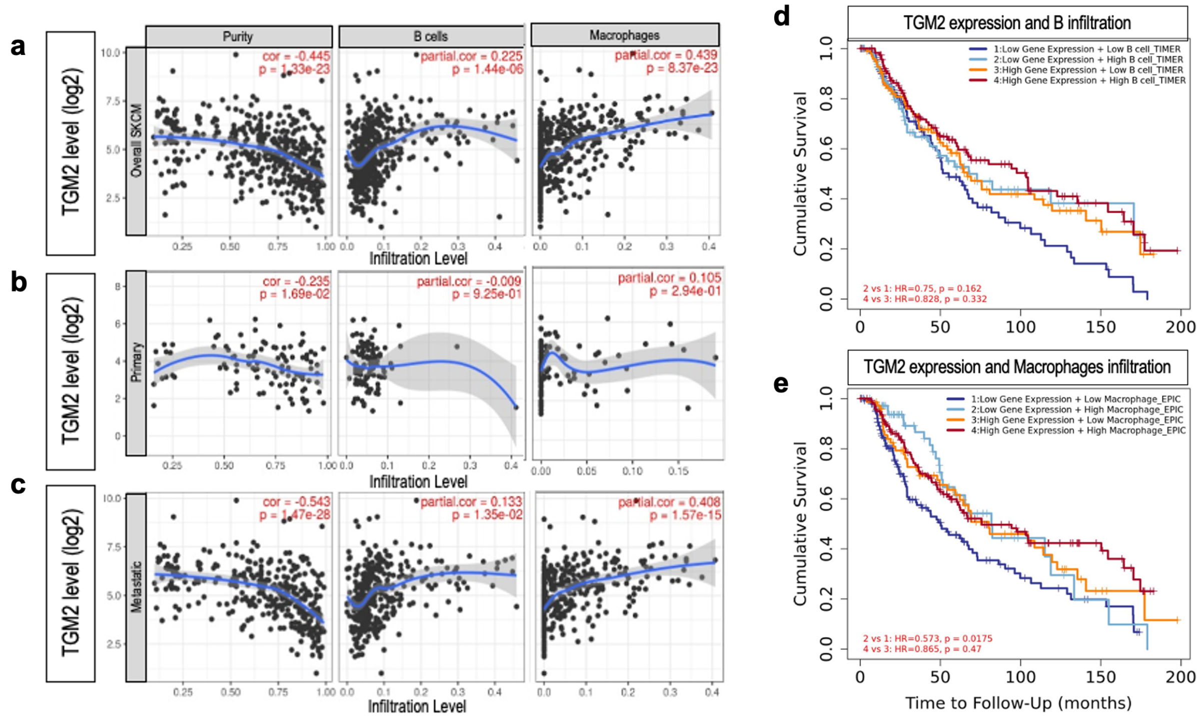

Figure S3.
